# Food safety-related practices among residents aged 18–75 years during the COVID-19 pandemic: a cross-sectional study in Southwest China

**DOI:** 10.1186/s12889-023-17608-1

**Published:** 2024-01-11

**Authors:** Zhourong Li, Ke Jiang, Shengping Li, Tiankun Wang, Huan Zeng, Manoj Sharma, Zumin Shi, Yong Zhao

**Affiliations:** 1 School of Public Health, Chongqing Medical University, Chongqing, 400016 China; 2 Research Center for Medicine and Social Development, Chongqing Medical University, Chongqing, 400016 China; 3 Research Center for Public Health Security, Chongqing Medical University, Chongqing, 400016 China; 4 Department of Social and Behavioral Health, School of Public Health, University of Nevada, Las Vegas (UNLV), Las Vegas, NV 89119 USA; 5 Department of Internal Medicine, Kirk Kerkorian School of Medicine, University of Nevada, Las Vegas (UNLV), Las Vegas, NV 89102 USA; 6 Human Nutrition Department, College of Health Sciences, QU Health, Qatar University, Doha, 2713 Qatar; 7 Chongqing Key Laboratory of Child Nutrition and Health, Children’s Hospital of Chongqing Medical University, Chongqing, 400014 China; 8 Nutrition Innovation Platform-Sichuan and Chongqing, School of Public Health, Chongqing Medical University, Chongqing, 400016 China

**Keywords:** Food safety-related practices, Southwest China, Gender, COVID-19 pandemic

## Abstract

**Background:**

Good food safety practices are essential to minimizing foodborne diseases. The present study explored the food safety-related practices of residents during the COVID-19 pandemic in Southwest China and identified the impacting factors.

**Methods:**

Residents aged 18–75 years from Guizhou, Yunnan, Sichuan, and Chongqing, China, were included in our study. The convenience sampling method was used to select participants, and face-to-face surveys were conducted in households and communities to collect data. Descriptive statistics including sociodemographic characteristics of respondents and weighted percentages were obtained and the log-binomial regression was used to evaluate the influencing factors associated with food safety-related practices.

**Results:**

Overall, 7,848 respondents were involved, with 97.5% efficacy. Disparities in food safety-related practices were observed between males and females, with the former performing poorer practices than the latter (70.5% vs. 68.0%, respectively). Notably, paying attention to nutrition labels when shopping for prepackaged foods was the worst practice. Age, ethnicity, region, occupation, education level, and income were identified as significant determinants of food safety-related practices. Moreover, in comparison to males, females were more likely to acquire pertinent knowledge from diverse sources, including social media, family members/ friends, books/ newspapers/ magazines, experts, and food sales staff (*p* < 0.05).

**Conclusions:**

Males performed inferior food safety-related practices than females during the COVID-19 pandemic in Southwest China. It is suggested that future food safety education programs should incorporate diverse targeted approaches, with emphasis on males. The role of mainstream media in promoting food safety practices should be expanded and prioritized in the forthcoming initiatives.

## Background

Food safety is closely associated with global public health and socio-economic development [1]. According to the World Health Organization (WHO) survey on food safety, unsafe food causes more than 200 diseases and an estimated 600 million cases, resulting in 420 thousand deaths and a loss of 33 million healthy life years (DALYs) [2]. The China Food Safety Development Report (2019) identified that microbiological contamination, over-range/ over-limit use of food additives, non-compliance with quality indicators, non-compliance with pesticide and veterinary drug residues, and heavy metal contamination as the five most significant food safety issues, posing severe health effects on humans, particularly on at-risk populations [3]. Mitigating the prevalence of foodborne diseases remains a major global public health challenge [4].

During the COVID-19 pandemic, heightened concerns regarding food safety, particularly associated with the processing, packaging, and transportation of agricultural products, have emerged as a significant public issue [5]. SARS-CoV-2, the pathogen of COVID-19, has demonstrated the potential to remain infectious for up to 14 days at 4 °C and has been identified as a possible source of transmission through imported frozen food [6–9]. A qualitative risk assessment revealed two possible exposure routes to SARS-CoV-2: consumption of contaminated animal products and the intake of cross-contaminated foods, used contact materials, or infected individuals who work in food preparation [10]. Consequently, ensuring food safety is imperative in mitigating the risk of both COVID-19 transmission and foodborne illness.

Poor food safety and personal hygiene are the primary risk factors for foodborne diseases [11, 12]. Food safety-related practices refer to the actions of individuals pertaining to the safety, preparation, consumption, and handling of food, aimed at preserving food quality and preventing contamination and foodborne illnesses [13]. Keeping clean, separating raw and cooked, cooking thoroughly, keeping food at safe temperatures, and using safe water and raw materials are the “five keys to safer food” developed by WHO that promote safe food handling and facilitate consumers to learn safe practices that guarantee food safety [10].

To date, various studies on food safety have employed the knowledge-attitude-practice (KAP) theory model, revealing a need for further promotion of food safety practices. A cross-sectional study conducted in Ethiopia showed that college students exhibited remarkably low levels of overall knowledge, practices, and attitudes toward food safety [14]. Research on food handlers indicated that satisfactory food safety knowledge translates into strict hygienic practices that need further improvement [1, 15–17]. A mixed-method study regarding COVID-19 prevention in Iran showed that the food safety practices of restaurant managers were acceptable [18]. However, these findings are often specific to study populations (e.g., food handlers, students, and consumers) and may lack overall generalizability. The Statistical Bulletin for China’s Health Care Development (2022) reported that a total of 5,493 outbreaks of foodborne illnesses were reported in China, with 32,334 cases and 117 deaths nationwide [19]. Southwest China, characterized by diverse catering cultures and regional nuances, has witnessed a notable increase in food safety concerns, particularly incidents of mushroom poisoning [20] over recent years. In 2022, mushroom poisoning resulted in 1,332 patients and 28 deaths nationwide, with a total case fatality rate of 2.1% [21]. Further research is imperative to promote safe food handling and mitigate the risk of foodborne illnesses. In this study, a cross-sectional study in Southwest China was conducted among residents aged 18–75 years during the COVID-19 pandemic to explore the food safety-related practices status and better understanding of its impact factors, to develop more effective and scientifically sound strategies for food safety.

## Methods

### Study design and sample

The cross-sectional study was carried out in Southwest China, including Guizhou, Yunnan, Sichuan, and Chongqing, from February 5 to May 10, 2021. A total of 252 screened and trained investigators from eight universities (Chongqing Medical University, Chengdu University of Traditional Chinese Medicine, Guizhou, and Kunming Medical University, etc.) were recruited before the survey. Paper questionnaires were mailed to each investigator. The convenience sampling method was used for conducting face-to-face surveys in households and communities. The survey required approximately 5–8 min to complete.

The inclusion criteria for the study were as follows: (a) aged 18–75 years; (b) local resident for at least 1 year; and (c) be able to understand and complete the questionnaires. The exclusion criteria were those unable to cooperate in completing the survey due to illness or other factors.

The sample size required for the study was estimated by the sample size calculation formula of the cross-sectional study: $$ n=\left(\frac{{{Z}_{\propto /2}}^{2}}{{d}^{2}}\right)*p(1-p)$$. Referring to the awareness rate of dietary nutrition knowledge among Chinese residents in 2015, the $$ p$$ was 0.0211, the margin of error $$ d$$ = 0.20 $$ p$$, and $$ {Z}_{\propto /2}$$ = 1.96. The calculated sample size was determined to be 1,450 for each region and 5,800 in total. Considering sampling error and invalid response, an additional 10.0% to20.0% was added to the estimated sample size, resulting in a final target sample of 6,380 to 6,960 individuals. After excluding 202 questionnaires with outliers and missing data. A total of 7,848 residents were included in our study.

Informed consent was obtained from all participants before the study. Our study was approved by the Ethics Committee of Chongqing Medical University on July 13, 2020. (Record number: 2,021,041).

### Data Collection

#### Instrument

The survey was initiated by the Chinese Nutrition Society, and the questionnaire was designed by the Chinese Nutrition Society Science Communication and Popularization experts. Additional entries pertaining to the dietary habits of Southwest China were included, and the final draft was formulated with the guidance of experts in the field of nutrition and health behavior. Based on the pilot survey, the Cronbach’s α coefficient was 0.825, which was greater than 0.80, while the Kaiser-Meyer-Olkin (KMO) measure was 0.859, indicating that the questionnaire had acceptable internal consistency and structural validity. The questionnaire consisted of two parts: social demographic characteristics (11 questions) and food safety-related practices (7 questions).

#### Social demographic characteristics

Social demographic characteristics included gender (Male/Female), age groups (18–44 years, 45–59 years, and 60–75 years) [22], height, weight, ethnicity (Han/Minority), residence (Rural/Urban), region (Guizhou Province/Yunnan Province/Sichuan Province/Chongqing Municipality), occupation (Workers, including farmer, fisherman, herdsman, plant worker, contractor, fitness coach, businessman; Students; White-collar, including teacher, doctor, nurse, officials, public servant, lawyer, manager, office clerk, reporter; and Others, freelancer, retiree, and other occupations), education (low, junior high school and below; medium, senior high school/ junior college; and high, college/bachelor’s degree and above), the average monthly income of the households (5000 RMB, 5000–9999 RMB, 10,000 RMB and above), channels for acquiring pertinent knowledge about food safety (Experts, Books/Newspapers/Magazines, Radio or TV, Family members or friends, Social media (Network, Public accounts, TikTok and its Chinese version Douyin [23], etc.), and Food sales staff).

#### Food safety-related practices

Food safety-related practices include the following questions. (1) Paid attention to nutrition labels when shopping for prepackaged foods, (2) Separated raw and cooked, (3) Frequency of dining out (including takeout, excluding home and canteen meals), (4) Frequency of handwashing before meals, (5) Frequency of wildlife consumption (such as pangolins, bats, and wild birds), (6) Frequency of making raw and cold ready-to-eat (RTE) food (excluding salad), (7) Frequency of following the news and common sense concerning the safety issues of wild fungi during the wild fungi picking season.

### Data process

The Body Mass Index (BMI, weight/height^2^) was calculated by self-reported height and weight [24] and divided into underweight (< 18.5 kg/m^2^), normal (18.5 kg/m^2^ ≤ BMI < 24.0 kg/m^2^), overweight (24.0 kg/m^2^ ≤ BMI < 28.0 kg/m^2^), and obese (BMI ≥ 28.0 kg/m^2^).

Likert scale responses were adopted for the seven questions on food safety-related practices. The responses to each question were divided into three categories, with scores assigned to each option for questions regarding good practices (question 1, 2, 4, 7) and poor practices (question 3, 5, 6). Scores for good practices ranged from 0 for “Never/Occasionally” to 2 for “Usually/Always”, while scores for poor practices were assigned in reverse order. The total scores for the questions above ranged from 0 to 14. Participants achieving scores above 50% (> 7) of the total score were considered to exhibit satisfactory practices. The higher score indicated better food safety-related practices.

### Statistical analysis

Descriptive statistics, including frequencies and percentages (%), were used to describe the categorical variables, while the continuous variables were described by mean ± standard deviation (SD). The analysis of variance (ANOVA) test assessed the age difference by gender. The Chi-square tests were adopted to compare the differences in food safety-related practices according to diverse social demographic characteristics and show the distribution of responses concerning food safety-related practices by gender. The log-binomial regression evaluated the correlation between demographic characteristics and food safety-related practices.

All data were entered into EpiData3.1, and STATA version 17.0 (STATA Corporation, College Station, TX, USA) was used for data analysis. Statistical significance was defined as *p* < 0.05 (two-sided). The channels for obtaining food safety knowledge were visualized using Graph Pad Prism software version 9.0.

## Results

### Demographic characteristics of the participants

Table 1 summarized the demographic characteristics of 7,848 eligible participants from Southwest China, demonstrating a questionnaire validity rate of 97.5%. The average age of the participants was 35.1 ± 13.9 years (ranging from 18 to 75 years). Most of the participants lived in urban areas. The number of Han (88.4%) far exceeded that of the minority (11.6%). Additionally, 49.3% of the participants had a high educational attainment. Workers took up the largest part than other occupations in this survey, accounting for 40.7%. Moreover, 41.5% of the participants’ households earned less than 5000 RMB a month. The results showed that all the demographic characteristics aside from the region and ethnicity varied in gender (*p* < 0.05).

**Table 1:** Distribution of basic sociodemographic characteristics by gender in Southwest China, 2021 (N = 7,848)

Variables	Total	Gender		*p* ^1^
		Male	Female	
N (%)	7,848	3,607 (46.0)	4,241 (54.0)	
**Age, mean (SD)**	35.1 (13.9)	36.0 (14.0)	34.3 (13.7)	< 0.001
**Age**				< 0.001
18–44	5,522 (70.4)	2,431 (67.4)	3,091 (72.9)	
45–59	1,891 (24.1)	952 (26.4)	939 (22.1)	
60–75	435 (5.5)	224 (6.2)	211 (5.0)	
**Ethnicity**				0.79
Han	6,940 (88.4)	3,186 (88.3)	3,754 (88.5)	
Minority	908 (11.6)	421 (11.7)	487 (11.5)	
**Residence**				< 0.001
Rural	2,981 (38.0)	1,466 (40.6)	1,515 (35.7)	
Urban	4,867 (62.0)	2,141 (59.4)	2,726 (64.3)	
**Region**				0.11
Guizhou	2,204 (28.1)	1,061 (29.4)	1,143 (27.0)	
Yunnan	1,378 (17.6)	627 (17.4)	751 (17.7)	
Sichuan	1,562 (19.9)	709 (19.7)	853 (20.1)	
Chongqing	2,704 (34.5)	1,210 (33.5)	1,494 (35.2)	
**Education**				< 0.001
Low^2^	2,380 (30.3)	1,138 (31.5)	1,242 (29.3)	
Medium^3^	1,600 (20.4)	832 (23.1)	768 (18.1)	
High^4^	3,868 (49.3)	1,637 (45.4)	2,231 (52.6)	
**Occupation**				< 0.001
Worker	3,193 (40.7)	1,684 (46.7)	1,509 (35.6)	
Student	1,800 (22.9)	716 (19.9)	1,084 (25.6)	
White collar	1,624 (20.7)	663 (18.4)	961 (22.7)	
Others	1,231 (15.7)	544 (15.1)	687 (16.2)	
**BMI**				< 0.001
Underweight	890 (11.3)	224 (6.2)	666 (15.7)	
Normal	4,953 (63.1)	2,200 (61.0)	2,753 (64.9)	
Overweight	1,652 (21.1)	957 (26.5)	695 (16.4)	
Obese	353 (4.5)	226 (6.3)	127 (3.0)	
**Income**				< 0.001
< 5000 RMB	3,256 (41.5)	1,402 (38.9)	1,854 (43.7)	
5000–9999 RMB	2,387 (30.4)	1,149 (31.9)	1,238 (29.2)	
≥ 10,000 RMB	2,205 (28.1)	1,056 (29.3)	1,149 (27.1)	

^1^Chi-square test showing distribution by gender across demographic characteristics.

^2^Junior high school and below.

^3^Senior high school/junior college.

^4^College/bachelor’s degree and above.

### Responses to questions of food safety-related practices by gender

Responses to each question of food safety-related practices during the COVID-19 pandemic between males and females are presented in Table 2. In our study, about 30.8% of participants exhibited fair food safety-related practices, with females reporting superior practices compared to males (32.0% vs. 29.5%).

**Table 2:** Responses to food safety-related practices by gender in Southwest China, 2021 (N = 7,848)

Variables	Total (%)	Male (%)	Female (%)	*p* ^1^
	7,848	3,607 (46.0)	4,241 (54.0)	
**Practices level**				0.017
Fair	2,419 (30.8)	1,063 (29.5)	1,356 (32.0)	
Poor	5,429 (69.2)	2,544 (70.5)	2,885 (68.0)	
**Questions**				
**(1) Paid attention to nutrition labels when shopping for prepackaged foods**				
Never/Occasionally	48.1	49.8	46.6	0.003
Sometimes	22.4	22.5	22.3	
Usually/Always	29.5	27.7	31.1	
**(2) Separated raw and cooked**				
Never/Occasionally	22.8	24.7	21.2	< 0.001
Sometimes	21.0	22.7	19.7	
Usually/Always	56.1	52.6	59.1	
**(3) Frequency of dining out (including takeout, excluding home and canteen meals)**				
Never/Occasionally	53.4	51.4	55.1	< 0.001
Sometimes	32.0	32.1	31.9	
Usually/Always	14.6	16.5	13.0	
**(4) Frequency of handwashing before meals**				
Never/Occasionally	14.3	17.1	11.9	< 0.001
Sometimes	17.1	19.5	15.1	
Usually/Always	68.6	63.4	73.0	
**(5) Frequency of wildlife consumption (such as pangolins, bats, and wild birds)**				
Never/Occasionally	90.1	87.2	92.6	< 0.001
Sometimes	6.8	9.0	5.0	
Usually/Always	3.0	3.8	2.4	
**(6) Frequency of making raw and cold ready-to-eat (RTE) food (excluding salad)**				
Never/Occasionally	46.4	47.4	45.5	0.10
Sometimes	31.6	30.4	32.6	
Usually/Always	22.0	22.2	21.9	
**(7) Frequency of following the news and common sense concerning the safety issues of wild fungi during the wild fungi picking season**				
Never/Occasionally	39.1	39.2	39.0	0.97
Sometimes	27.2	27.0	27.3	
Usually/Always	33.7	33.7	33.7	

^1^Chi-square test showing the distribution of the response of food safety-related practices by gender

During the COVID-19 pandemic, our study indicated that 49.8% and 46.6% of males and females hardly paid attention to nutrition labels when shopping for pre-packaged foods (*P* < 0.01). More than half of males (52.6%) and females (59.1%) often separated raw and cooked (*P* < 0.001). Approximately half of participants rarely dined out, with 51.4% being males and 55.1% being females (*P* < 0.001). Moreover, 73.0% of females often washed their hands before meals, while 63.4% were males (*P* < 0.001). A significant majority of males (87.2%) and females (92.6%) rarely consumed wildlife (*P* < 0.001). The frequency of hardly making raw and cold RTE food exhibited no statistical difference between males and females (47.4% vs. 45.5%, *P* = 0.10), together with the frequency of often followed the news and common sense concerning the safety issues of wild fungi during the wild fungi picking season (33.7% vs. 33.7%, *P* = 0.97).

### Factors associated with food safety-related practices

The log-binomial regression analyses were used to identify the factors influencing food safety-related practices in different gender subgroups (Table 3) and underscored a significant association between food safety-related practices level and age, ethnicity, region, occupation, education, and income (*P* < 0.05).

**Table 3:** Log-binomial regression of food safety-related practices level in Southwest China (N = 7,848)

Variable	Total		Male		Female	
	OR	95%*CI*	OR	95%*CI*	OR	95%*CI*
	**Fair vs. Poor** ^1^		**Fair vs. Poor**		**Fair vs. Poor**	
**Age**						
18–44 (Ref)	1		1		1	
45–59	0.93	(0.82–0.97) **	0.94	(0.89–0.99) *	0.91	(0.86–0.97) **
60–75	1.05	(0.99–1.11)	1.05	(0.97–1.13)	1.05	(0.96–1.14)
**Ethnicity**						
Han (Ref)	1		1		1	
Minority	0.92	(0.87–0.97) **	0.93	(0.86-1.00) *	0.92	(0.85–0.99) *
**Residence**						
Rural (Ref)	1		1		1	
Urban	0.97	(0.94-1.00)	1.01	(0.96–1.05)	0.95	(0.91–0.99) *
**Region**						
Guizhou (Ref)	1		1		1	
Yunnan	0.83	(0.79–0.88) ***	0.87	(0.81–0.93) ***	0.80	(0.74–0.86) ***
Sichuan	0.98	(0.94–1.03)	1.01	(0.95–1.07)	0.96	(0.91–1.03)
Chongqing	1.03	(1.00-1.07)	1.02	(0.96–1.07)	1.05	(1.00-1.11) *
**Occupation**						
Worker (Ref)	1		1		1	
Student	1.07	(1.02–1.12) **	1.07	(1.00-1.14)	1.07	(1.00-1.15) *
White collar	0.90	(0.86–0.95) ***	0.93	(0.86-1.00)	0.89	(0.82–0.96) **
Others	0.99	(0.95–1.04)	1.03	(0.97–1.09)	0.97	(0.91–1.03)
**Education**						
Low (Ref)	1		1		1	
Medium	0.90	(0.86–0.94) ***	0.92	(0.86–0.97) **	0.87	(0.82–0.94) ***
High	0.89	(0.85–0.93) ***	0.88	(0.83–0.95) ***	0.88	(0.82–0.94) ***
**BMI**						
Normal (Ref)	1		1		1	
Thinness	1.03	(0.99–1.08)	0.99	(0.90–1.08)	1.06	(1.00-1.12) *
Overweight	0.98	(0.94–1.02)	0.97	(0.93–1.03)	0.96	(0.91–1.02)
Obese	1.03	(0.96–1.10)	1.02	(0.94–1.11)	1.02	(0.92–1.14)
**Income**						
< 5000RMB (Ref)	1		1		1	
5000–9999 RMB	0.96	(0.93-1.00) *	0.94	(0.89–0.99) *	0.97	(0.92–1.02)
≥ 10,000 RMB	0.97	(0.94–1.01)	0.97	(0.92–1.02)	0.97	(0.92–1.02)

^1^Fair food safety-related practices level as reference

OR, odds ratio; CI, confidence interval; *P < 0.05, **P < 0.01, ***P < 0.001

Subgroup analyses suggested that both males and females aged 45–59 years exhibited superior food safety-related practices compared to those aged 18–44 years. The odds ratio (OR) is 0.94 of males (95%CI: 0.89–0.99) and 0.91 of females (95%CI: 0.86–0.97), respectively. Notably, minority women had a higher level of practice than Han individuals (OR = 0.92, 95%CI:0.85–0.99). Urban-residing females exhibited better food safety-related practices than their rural counterparts (OR = 0.95, 95%CI: 0.91–0.99). Participants from Yunnan had significantly fairer food safety-related practices than those from Guizhou (OR = 0.83, 95%CI:0.79–0.88). Taking workers as a reference, the results indicated that schoolgirls had a poorer food safety-related practice level (OR = 1.07, 95%CI:1.00-1.15), while white collar females had relatively superior practices (OR = 0.89, 95%CI:0.82–0.96), although no such relation was observed among males. Education displayed a significant positive correlation with food safety-related practices level (*P* < 0.01), and those with a medium (OR = 0.69, 95%CI:0.59–0.80) or high level of education (OR = 0.67, 95%CI:0.57–0.79) were expected to perform better practices compared to those with a junior high school education and below. However, BMI showed no significant correlation with food safety-related practices level by gender.

### Channels for obtaining relevant knowledge of food safety

**Fig. 1:**
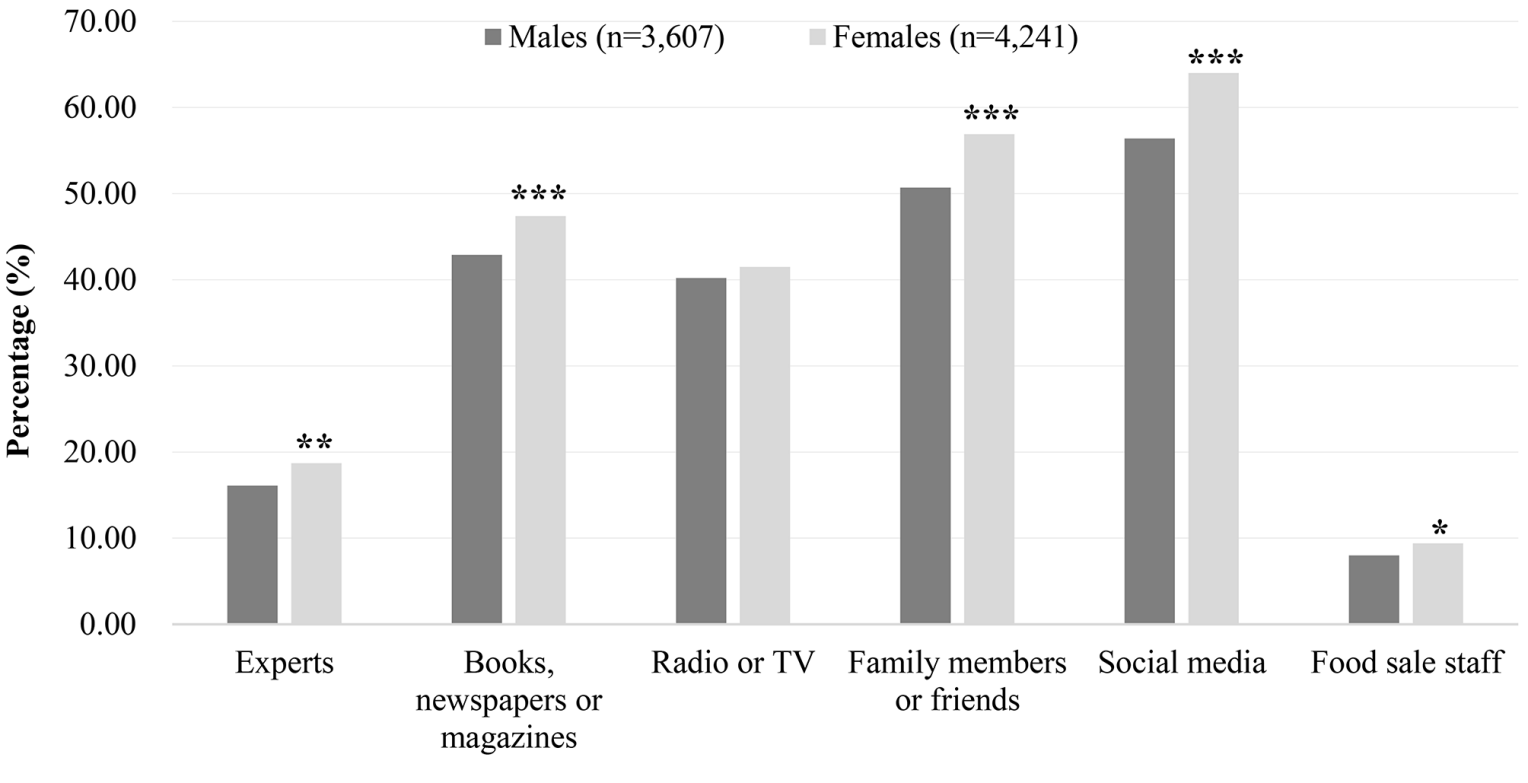
Channels for obtaining relevant knowledge of food safety, Southwest China, 2021 *P < 0.05, **P < 0.01, ***P < 0.001

Figure 1 demonstrated the proportion of males and females acquiring pertinent knowledge of food safety on six channels. In general, the predominant channels for both genders to obtain relevant knowledge were social media, followed by family members/friends, whereas obtaining information from food sales staff was the least common way. Females were more likely to obtain relevant knowledge from social media, family members/friends, books/newspapers/magazines, experts, and food sales staff, compared to males. Nevertheless, no statistical difference was observed in the channels for acquiring relevant knowledge from radio/TV by gender.

## Discussion

Given the significance of promoting food safety practices and shedding some light on its contributing factors, this large-scale cross-sectional study conducted during the COVID-19 pandemic revealed that merely 30.8% of Southwest China residents exhibited fair food safety-related practices, and males performed inferior practices compared to females. This highlighted the need for improvements, particularly in the aspect of paying attention to nutrition labels when purchasing prepackaged foods. Notably, individuals aged 45–59 years, minority, from Yunnan province, white collar, those with medium or high education level exhibited superior food safety-related practices in comparison to their counterparts. Conversely, students reported poorer practices than workers. In addition, whether males or females, social media platforms (Web, Public accounts, and TikTok) emerged as the primary channels for acquiring relevant knowledge about food safety.

During the COVID-19 pandemic, the public attached more importance to food safety. Proper diet and nutrition were the cornerstones of immunity against infection. Forbidding the purchase and consumption of wildlife, washing hands regularly, separating raw and cooked, cooking thoroughly, and reducing dining out are the core messages to recommend [10, 25]. The second stage of a nutrition survey conducted in post-lockdown China indicated that less than half of the participants adhered to the recommended dietary practices, which is in accordance with the findings of our study. However, the proportion of often preparing raw and cooked food separately was higher than that in our study (68.1% vs. 56.1%) [26]. A cross-sectional study in China also observed that people were more likely to eat on individual plates, use serving chopsticks and separate plates and utensils for raw and cooked food during the post-COVID-19 lockdown period as compared to the pre-COVID-19 period, which are crucial in preventing the cross-contamination of bacteria or other microorganisms [27, 28]. Raw products, retail-level RTE foods, frozen foods, and mushrooms were identified as potential sources of bacteria, underscoring the importance of thorough cooking, whereas our study revealed that over one-fifth of residents often make RTE foods, emphasizing the necessity for promoting healthy cooking practices [29].

With regards to dining behavior, our study found that 53.4% of residents hardly dined out during the COVID-19 pandemic, which was lower than an online survey in Jordan. Tareq et al. claimed that over 70.0% of university students dined out less than formerly, reducing eating and gathering with their friends and family members [30]. A similar pattern was observed in Qatar, where approximately half of people reported cooking and preparing food more frequently, choosing to eat more often with immediate family members during the COVID-19 period [31]. According to Nonato et al. [32], street food gastronomy can lead to the development of foodborne diseases and chronic diet-related diseases. Consequently, the public’s perception of COVID-19 risks about the food itself and the imposed dining restrictions appear to be the primary influencing factors [33].

Nutrition labels on prepackaged foods are considered as a highly credible source to obtain accurate nutrition information, such as protein, carbohydrates, total fat, sodium, calories, and shelf life, which properly guide consumers to choose healthier and more nutritious food [34–36]. Surprisingly, paying attention to nutrition labels when shopping for prepackaged foods was the least prioritized behavior. Females exhibited better practices than males, potentially attributed to their heightened understanding of nutrition labels, greater attention to nutrition and health, and superior dietary habits [37, 38]. Urgent measures are warranted to enhance education and awareness regarding nutrition labels on prepackaged foods, with a specific focus on increasing residents’ awareness, particularly among males.

The potential of the animal-human interface as the primary source of emerging zoonotic diseases was reaffirmed at the onset of the COVID-19 outbreak [39]. Our study revealed that most residents refrained from consuming wildlife during this period, contributing to potential benefits in biodiversity conservation [39, 40]. Before the widely used effective treatment or vaccine for COVID-19, hand disinfection and mask-wearing were regarded as cost-effective measures for preventing transmission [41, 42]. Simultaneously, favorable hand hygiene practices are the key to preventing fecal-oral transmission of foodborne diseases and ensuring food safety. Given that food can serve as a vehicle for transmitting pathogens like norovirus, cholera, Escherichia coli, and Hepatitis A virus, adherence to good hand hygiene practices can mitigate the risk of poisoning [43–45]. An outbreak of norovirus in an Austrian boarding school highlighted the imperative for strict adherence to hand hygiene guidelines among food handlers [46]. Residents should strengthen personal hygiene measures-washing hands with soap and water for a minimum of 20 s, in accordance with WHO guidelines. Although over half of the survey respondents reported handwashing before meals, hand hygiene remains a concern. A quasi-experimental study examining the impact of health and food safety training interventions on restaurant food handlers demonstrated that implementing such interventions can effectively enhance community health [47]. It is recommended to advocate for community outreach and educational interventions to enhance compliance with pre-meal hand hygiene behavior.

The food safety-related practices significantly differed across gender, with females performing better practices than males. This gender contrast may be attributed to the traditional roles in Chinese households, where wives assume central responsibilities in daily kitchen activities, food preparation, and cleaning, as well as the tradition for mothers to pass down their abundant food-related experience to their daughters [30]. Concurrently, women traditionally shoulder crucial duties in food procurement, preparation, and preservation [48].A study spanning 1991 to 2015 on adherence to recommended diets in China indicated that females outperformed males in compliance and diet quality [49]. These findings suggested that women may possess a heightened awareness of food safety practices in their daily routines. Future efforts should focus on empowering women through information and communication technologies, social support, and familial rights, enabling them to get timely information from food nutrition and security experts, which can play a pivotal role in promoting food security and fostering healthy eating habits within their families [50].

Our findings indicated that middle-aged individuals performed superior practices than both younger and elderly counterparts when it comes to food safety. The fast-paced lifestyle of millennials, marked by a preference for frequent dining out and takeout orders, contrasts with the middle-aged demographic, which tends to have more time for home food preparation and greater experience with food safety considerations [51]. Moreover, the elderly may hold deeply ingrained beliefs and be heavily influenced by traditional thinking, potentially emphasizing personal experience over updated safety practices. This may pose challenges in modifying misconceptions related to food safety behaviors among the elderly. Our study revealed a clear correlation between educational attainment and adherence to good practices in food safety, in line with previous studies [15, 16, 52, 53]. Thus, it is essential to consider the education levels when delivering food safety education. Pertaining to occupation, our study demonstrated that students exhibited inadequate food safety practices, aligning with the findings of Jember et al. [14]. Students may eat in the canteen or order takeaways more frequently and demonstrate lower engagement in food safety practices in their daily lives. It is imperative to contemplate the integration of food education, particularly emphasizing food safety practices, within the school curriculum. The implementation of concise courses and lectures covering essential food safety principles is strongly advocated [30]. Pioneering a novel model wherein students take the lead in promoting family-wide food safety practices could signify a constructive and forward-thinking initiative. Additionally, local governing bodies should proactively champion health education initiatives to steer residents toward cultivating a rational and well-informed outlook on food safety.

Regarding the channels for acquiring pertinent knowledge of food safety by gender. It was noted that social media has emerged as the primary platform for obtaining information on nutrition and health, which can be attributed to the rapid advancements in internet technology, coupled with the widespread use of mobile devices [54, 55]. Gender differences were noticeable, with females paying more attention to health information than their counterparts. However, a cohort study of worksite wellness center members showed no meaningful gender differences concerning self-efficacy for maintaining a healthy diet [56]. In the future, there is an anticipation that inclusive food safety initiatives designed for both genders will be implemented, with social media platforms such as TikTok and network channels playing a pivotal role in their execution.

Our study has some limitations. Firstly, the geographical representation of the sample population is constrained, encompassing only four regions in Southwest China, and utilizing a convenience sampling method. Consequently, the generalizability of the findings to other parts of the country may be limited. Secondly, despite rigorous quality control measures implemented during the face-to-face survey, it is essential to acknowledge the potential impact of recall bias and self-reported practices on data quality. Furthermore, research on food safety in China is still in its early stages, characterized by a combination of aggregate data and small case studies, which may not provide an accurate picture of the actual risks to which populations or localities are exposed. Lastly, the capacity to draw direct causal inferences is limited when using cross-sectional survey data. More comprehensive and detailed research is required to delve into the intricate factors influencing food safety practices.

## Conclusions

To sum up, this study with a large-scale population-based sample was a representative study of exploring the food safety-related practices status and the impacting factors during the COVID-19 pandemic in Southwest China. Males performed inferior food safety-related practices than females. It is suggested that future food safety education programs should incorporate diverse targeted approaches, with emphasis on males. Age, ethnicity, gender, occupation, and education were the primary contributing factors. Notably, social media emerged as the predominant channel for obtaining pertinent information on food safety. These insights provide valuable reference points for governmental, educational, and other relevant entities to formulate more precise, scientific, and efficacious strategies tailored to specific demographic groups. The role of mainstream media in promoting food safety practices should be expanded and prioritized in the forthcoming initiatives.

## Data Availability

The datasets used and/or analyzed during the current study are available from the corresponding author upon reasonable request.
